# Relationship between cardiovascular health score and year-to-year blood pressure variability in China: a prospective cohort study

**DOI:** 10.1136/bmjopen-2015-008730

**Published:** 2015-10-26

**Authors:** Shasha An, Minghui Bao, Yang Wang, Zhifang Li, Wenyan Zhang, Shuohua Chen, Junjuan Li, Xinchun Yang, Shouling Wu, Jun Cai

**Affiliations:** 1 Department of Cardiology, Chaoyang Hospital, Capital Medical University, Beijing, China; 2 Department of Cardiology, Kailuan Hospital, Hebei United University, Tangshan, China; 3 Department of Nephrology, Kailuan Hospital, Hebei United University, Tangshan, China

**Keywords:** PUBLIC HEALTH

## Abstract

**Objectives:**

On the basis of cardiovascular health factors and behaviours, the American Heart Association proposed the Cardiovascular Health Score (CHS). It has been widely used to estimate the cardiovascular health status of individuals. The aim of this study was to investigate the relationship between CHS and year-to-year blood pressure variability (BPV).

**Design:**

Prospective cohort study.

**Settings:**

We stratified participants into two groups by gender: first group, female group; second group, male group. The relationship between CHS and year-to-year blood pressure variability were analysed.

**Participants:**

A total of 41 613 individuals met the inclusion criteria (no history of stroke, transient ischaemic attack, myocardial infarction, malignant tumour or atrial fibrillation) and had complete blood pressure data.

**Results:**

The coefficient of the variation of systolic blood pressure (SCV) was 8.33% in the total population and 8.68% and 8.22% in female and male groups, respectively (p<0.05). Multivariable linear regression analysis revealed that higher CHS was inversely associated with increasing year-to-year BPV, which persisted after adjusting for baseline systolic blood pressure and other risk factors. Each SD increase in CHS could lead to a 0.016SD decrease in SCV (p<0.05).

**Conclusions:**

In summary, CHS was inversely related to year-to-year BPV, which suggested that a healthy lifestyle may contribute to better blood pressure management.

**Strengths and limitations of this study**

- Our present work is the first research aimed at estimating the relationship between cardiovascular health score and long-term blood pressure variability to date.
- The present study observed an inverse relationship between cardiovascular health score and year-to-year blood pressure variability and further supported that healthier lifestyle might contribute to better blood pressure management.
- We did not adhere perfectly to all of the seven health metrics for practical reasons. For example, we used the amount of salt intake as a surrogate of ideal diet, which might underestimate the effects of diet on blood pressure variability.

## Introduction

Growing evidence suggests that blood pressure (BP) values alone may not fully explain the pathophysiological relationship between BP and adverse cardiovascular effects of hypertension. Post hoc analyses of clinical trials and observational studies indicate that blood pressure variability (BPV)—that is, the extent of BP fluctuation over a time period—is associated with cardiovascular and cerebrovascular events.^1–4^ Several studies have reported relationships between BPV and cardiovascular events,^5^ ^6^ mortality^1^ and end-organ damage.^7–9^

BPV is characterised by short-term fluctuations (eg, beat-to-beat, minute-to-minute, hour-to-hour, day-to-night and within-visit) and long-term fluctuations (eg, day-to-day, week-to-week, month-to-month and year-to-year). Compared with short-term BPV, long-term BPV is a much more reproducible phenomenon.^10^ Recent data suggest that visit-to-visit variability over longer follow-up periods has greater prognostic value than average BP or short-term variability,^11^ ^12^ leading to increased interest in the prognostic importance of long-term BPV.

In 2010, the American Heart Association (AHA) first proposed a definition of cardiovascular health behaviours and health factors.^13^ It comprises seven metrics—four health behaviours (smoking, diet, physical activity and body weight) and three health factors (plasma glucose, cholesterol and BP)—which are used to categorise individuals into ‘poor’, ‘intermediate’ and ‘ideal’ groups. To estimate individual-level changes in cardiovascular health factors and behaviours, Huffman^14^ created the AHA Cardiovascular Health Score (CHS), which includes all seven cardiovascular health behaviours and health factors (poor, 0 points; intermediate, 1 point; ideal, 2 points; total scale: 0–14 points).

Several studies have demonstrated that improvements in cardiovascular health behaviours and health factors, hsCRP^15^ and carotid artery intima-media thickness^16^ ^17^ are associated with a gradually decreasing incidence of cardiovascular and cerebrovascular events.^18–23^ It was recently proposed that this association could be partly attributed to favourable impacts on CVD biomarker levels and subclinical diseases.^24^ CHS is a protective factor against cardiovascular and cerebrovascular events, and could potentially have a protective effect related to year-to-year BPV. Here, we performed a prospective cohort study and analysed the impact of different CHSs on year-to-year BPV based on the Kailuan cohort (ChiCTR-TNC-11001489).

## Materials and methods

The written informed consents were obtained from all participants in this study.

### Study participants

This prospective cohort study was based on the Kailuan Community in Tangshan, a large modernised city on the Bohai coast. The Kailuan Community is a fully functional community owned and administrated by the Kailuan Group Corporation. Its healthcare is provided by 11 hospitals. The first health examination for in-service and retired workers was performed in 2006–2007. The second (2008–2009), third (2010–2011) and fourth (2012–2013) health examinations involved the same investigations and measurements performed by the same medical staff in the same location, on the same workers who participated in the first examination and following the same sequence.

### Inclusion criteria and exclusion criteria

The inclusion criteria were as follows: completion of all four health examinations, providing complete information regarding cardiovascular health behaviours and health factors, and giving signed informed consent to participate in the study. Exclusion criteria were as follows: history of stroke (except for lacunar infarction), myocardial infarction, malignant tumour or atrial fibrillation; or missing BP data from any of the four examinations.

### Data collection

#### Epidemiological questionnaire

The questionnaire was completed by individuals and then verified by research doctors. The questionnaire items covered general information (birth date, gender, occupation, economic status, educational level, labour intensity, etc), health information (history of hypertension, diabetes, hyperlipidaemia, trauma, heart disease, stroke, other vascular diseases, malignant tumour, infectious diseases, etc), medication situation, family health information and diet and lifestyle (eg, exercise, time of work and rest, smoking history and drinking history).

#### Physical examination

The physical examination adopted standard measurements performed by trained doctors. Height and weight were measured using a corrected RGZ-120 scale (accurate to 0.1 cm and to 0.1 kg, respectively), with the participants in thin clothing and without shoes or hats. Waist and hip circumference were measured with inelastic tape (accuracy, 1 mm), with the individuals standing vertically with their feet separated by 30–40 cm and arms dropped naturally, continuing normal breathing and without tucking up. Waist circumference measurement was taken at the end of expiration, with the measuring tape placed horizontally between the hip and the narrowest part of the waist. For hip circumference measurement, the tape was placed on the widest part of the hip. The measurement error was required to be <0.1 cm.

BP was measured between 7:00 and 9:00. Individuals were asked to refrain from smoking and drinking tea or coffee for more than 30 min and to sit and rest for 15 min prior to measurement. During BP measurement, individuals sat with their arms and feet flat, and their upper arms at the height of their heart. Right brachial artery BP was measured by a corrected mercury sphygmomanometer with an appropriate sized cuff. Systolic blood pressure (SBP) was recorded on hearing the phase I Korotkoff sound. Diastolic blood pressure (DBP) was recorded on hearing the phase V Korotkoff sound. Sitting BP was measured three times, with a 30 s interval. If two measurements differed by >5 mm Hg, BP was remeasured. Final BP was calculated as the mean of three measurements.

#### Laboratory data collection

Biochemical measurements included fasting plasma glucose (FBG), triglycerides (TG), total cholesterol (TC), high-density lipoprotein cholesterol (HDL-C) and low-density lipoprotein cholesterol (LDL-C). Blood samples were collected from the antecubital vein in the morning after an overnight fast, and transferred into EDTA-containing vacuum tubes. FBG was measured by the hexokinase method. TC and triglycerides were measured enzymatically (interassay coefficient of variation <10%; Mind Bioengineering Co Ltd, Shanghai, China). All biochemical variables were measured using an automatic analyser (Hitachi 7600 automatic analyser) at the central laboratory of the Kailuan General Hospital.

### Relevant definitions

#### Health behaviours

Categories of cigarette smoking were as follows: ideal, never smoker; intermediate, used to smoke but not now; poor, current smoker. The body mass index (BMI) limits chosen were consistent with previously proposed overweight and obesity reference standards for Chinese adults.^25^ BMI categories were as follows: ideal, <24 kg/m^2^; intermediate, 24–27.9 kg/m^2^; poor, ≥28 kg/m^2^. Physical activity categories were as follows: ideal, >3 times per week for >30 min each time; intermediate, occasional exercise; poor, no physical activity. In contrast, the AHA defines ideal exercise as >5 times per week for >30 min each time. Salt intake greatly impacts cardiovascular disease in the Chinese population, and thus our questionnaire included salt intake rather than vegetable intake as a health behaviour. Diet categories were as follows: ideal, light salt; intermediate, moderate salt; poor, heavy salt.

#### Health factors

Untreated TC<5.18 mmol/L was considered ideal, TC of 5.18–6.21 mmol/L or treated TC<5.18 mmol/L was intermediate, and TC≥6.22 mmol/L was poor. Untreated SBP<120 mm Hg and DBP<80 mm Hg were considered ideal, SBP of 120–139 mm Hg or DBP 80–89 mm Hg or treated BP<140/90 mm Hg was considered intermediate, and SBP≥140 mm Hg or DBP ≥90 mm Hg was considered poor. Untreated FBG of <6.1 mmol/L was ideal, FBG 6.1–6.9 mmol/L or treated FBG<6.1 mmol/L was considered intermediate, and FBG≥7.0 mmol/L was considered poor.

Considering that the aim of this study is to estimate the relationship between CHS and BPV, we excluded BP from the seven metrics and estimated the relationship between CHS with the remaining six metrics and BPV. To better assess their relationship, we adopted the scoring system based on six cardiovascular health behaviours and health factors (poor, 0 points; intermediate, 1 point; ideal, 2 points; total scale: 0–12 points).

#### Blood pressure variability

Year-to-year BPV was described as the coefficient of the variation of BP (CV, calculated as SDBP/average BP×100%). Yearly mean SBP was calculated as the mean of the three measurements from each health examination (2006–2007, 2008–2009, 2010–2011 and 2012–2013). Then the yearly mean and SD of SBP were calculated on the basis of the 4 yearly SBP values. The CV of SBP was recorded as SCV, and the CV of DBP was recorded as DCV.

### Statistical methods

Data were entered in the terminal of each hospital and then uploaded to the computer room of the Kailuan General Hospital for storage in an Oracle 10.2 g database. SPSS V.13.0 statistical software was utilised for statistical analysis. Normally distributed measurement data were recorded as mean±SD. Intergroup comparisons were performed by t test. Abnormally distributed measurement data were converted to normally distributed data by logarithmic transformation and then analysed by variance analysis (t test). A categorical variable was recorded as n (%). Inter-group comparisons were performed by Pearson’s χ^2^ test. Multivariable linear regression models (enter method) were used to analyse the influencing factors of year-to-year BPV. Considering the fact that there might be collinearity between independent variables, we analysed the variance inflation factor (VIF) of each independent variable. If there was a great collinearity (VIF>10), we removed the variable with great collinearity and reperformed the multivariable linear regression. Factors influencing year-to-year BPV were analysed using a logistic regression model and p<0.05 (bilateral) was considered statistically significant.

## Results

Among the 101 510 workers who participated in the 2006–2007 health examination, 5568 were excluded: 1162 for lack of baseline BP data, and the rest for medical history (1316 myocardial infarction, 2353 stroke, 327 malignant tumour, and 410 atrial fibrillation). Of the remaining 95 942 individuals, 45 731 finished the second, third and fourth health examinations, among whom 2470 lacked complete BP data and 1 648 lacked indices of the seven cardiovascular health behaviours and factors. Finally, a total of 41 613 participants were included in the final statistical analysis.

### Baseline characteristics of participants

The baseline characteristics of participants are showed in table 1. Within the observation cohort, 31 647 participants were male and 9966 were female. Their ages ranged from 18 to 94 years (47.9±11.4 years). A total of 15 846 participants were diagnosed with hypertension (SBP>140 mm Hg or DBP>90 mm Hg or receiving antihypertensive therapy). A total of 12 236 participants did not take antihypertensive drugs, 1917 of them were in the female group (19.2%) and 10 319 in the male group (32.6%). The age, SBP, DBP, BMI, FBG, lgTG and proportion of alcohol consumption of males were higher than those of females (p<0.05). However, the lghsCRP and proportion of antihypertensive drug usage of males were lower than those of females (p<0.05).

**Table 1:** Baseline characteristics of participants

	Total (n=41 613)	Female (n=9966)	Male (n=31 647)	p Value
Age, years	47.9±11.4	47.4±10.9	48.0±11.6	<0.001
SBP, mm Hg	128.0±19.6	123.1±20.2	129.6±19.2	<0.001
DBP, mm Hg	82.6±11.4	78.9±10.8	83.7±11.3	<0.001
BMI, kg/m^2^	25.10±3.48	24.58±3.74	25.26±3.38	<0.001
lg TG	0.13±0.27	0.08±0.25	0.12±0.27	<0.001
lg hsCRP	−0.16±0.67	−0.13±0.67	−0.17±0.67	<0.001
Alcohol consumption, n (%)	8470 (20.4)	77 (0.8)	8393 (26.5)	<0.001
Antihypertensive drug usage, n (%)	3610 (8.70)	1010 (10.1)	2600 (8.2)	<0.001

1 mm Hg=0.133 kPa; BMI, body mass index; DBP, diastolic blood pressure; lg hsCRP: logarithm of high-sensitivity C reactive protein; lg TG: logarithm of triglycerides; SBP, systolic blood pressure.

### CV in different gender groups

The CV of different gender groups is shown in table 2. SCV and DCV were 8.33% and 8.37%, respectively, for the total population. SCV of the male group was higher than that of the female group (p<0.05); however, DCV of the male group was lower than that of the female group (however, there was no significant difference, p=0.616).

**Table 2:** CV of different gender groups

	Total (n=41 613)	Female (n=1657)	Male (n=26 247)	p Value
SCV, (%)	8.33±4.08	8.68±4.26	8.22±4.02	<0.001
DCV, (%)	8.37±4.03	8.38±3.39	8.36±4.06	0.616

DCV, coefficient of the variation of diastolic blood pressure; SCV, coefficient of the variation of systolic blood pressure.

### Multivariable-adjusted linear regression analysis with each SD increase in SCV as a dependent variable

Multivariable-adjusted linear regression analysis with each SD increase in SCV as a dependent variable is shown in table 3. Each SD increase of SCV was regarded as the dependent variable. Independent variables included each SD increase of CHS, age, male gender, baseline SBP, lghsCRP, lgTG, alcohol consumption and proportion of antihypertensive drug use. Model 1, with each SD increase of CHS, age and male gender as independent variables. The results suggested that each SD increase in CHS could lead to a 0.027SD decrease in SCV (p<0.05). Model 2, based on the model 1 results, we adjusted for baseline SBP. The results suggested that each SD increase in CHS could lead to a 0.020SD decrease in SCV (p<0.05). Model 3, based on the model 2 results, we adjusted for lghsCRP, lgTG, alcohol consumption and proportion of antihypertensive drug usage. The results suggested that each SD increase in CHS could lead to a 0.016SD decrease in SCV (p<0.05).

**Table 3:** Multivariable-adjusted linear regression analysis with each SD increase in SCV as a dependent variable

Influencing factors	β	SE	β	P	95% CI of B	VIF
Model 1						
CHS	−0.027	0.005	−0.027	0.000	−0.037 to −0.017	1.093
Age	0.014	0.000	0.156	0.000	0.013 to 0.014	1.000
Male	−0.141	0.012	−0.060	0.000	−0.164 to −0.117	1.094
Model 2						
CHS	−0.020	0.005	−0.020	0.000	−0.03 to −0.01	1.121
Age	0.012	0.000	0.140	0.000	0.011 to 0.013	1.129
Male	−0.150	0.012	−0.064	0.000	−0.174 to −0.127	1.103
SBP	0.002	0.000	0.047	0.000	0.002 to 0.003	1.176
Model 3						
CHS	−0.016	0.006	−0.016	0.004	−0.027 to −0.005	1.295
Age	0.012	0.000	0.137	0.000	0.011 to 0.013	1.173
Male	−0.145	0.012	−0.062	0.000	−0.169 to −0.121	1.154
SBP	0.002	0.000	0.042	0.000	0.002 to 0.003	1.276
lgTG	0.005	0.019	0.001	0.802	−0.033 to 0.042	1.131
lghsCRP	0.014	0.007	0.009	0.069	−0.001 to 0.028	1.056
Alcohol consumption	0.005	0.014	0.002	0.692	−0.021 to 0.032	1.140
Proportion of antihypertensive drug usage	0.069	0.018	0.020	0.000	0.033 to 0.105	1.140

Model 1, with each SD increase in CHS, age and male gender as independent variables. Model 2, adjusted for baseline SBP on the basis of Model 1. Model 3, adjusted for lgTG, lghsCRP, alcohol consumption and proportion of antihypertensive drug usage on the basis of Model 2.

CHS, Cardiovascular Health Score; lg TG: logarithm of triglycerides; lghsCRP, logarithm of high-sensitivity C reactive protein; SBP, systolic blood pressure; SCV, coefficient of the variation of systolic blood pressure; VIF, variance inflation factor.

## Discussion

Our results showed that year-to-year BPV decreased with increasing CHS. Each SD increase in CHS could lead to a 0.027SD decrease in SCV (p<0.05). Although the CV could attenuate the influence of the mean BP to some extent, it still influenced by average BP.^11^ Therefore, we adjusted for baseline SBP to reduce the impact of the mean BP value. The results indicated a strong inverse relationship between CHS and year-to-year BPV. Each SD increase in CHS could lead to a 0.020SD decrease in SCV (p<0.05). Since previous studies have shown that year-to-year BPV is influenced by age,^11^ gender^1^ and other factors, we adjusted multiple influencing factors as well. After adjusting for these factors, our multivariable regression analysis continued to indicate that each SD increase in CHS could lead to a 0.016SD decrease in SCV (p<0.05). These findings confirmed that better cardiovascular health behaviours and health factors could lead to a lower year-to-year BPV.

Why does CHS have such a relationship with year-to-year BPV? This, indeed, is a very complex issue. Although the short-term BPV might be largely influenced by central and reflex autonomic modulation,^26^ ^27^ the elastic properties of arteries,^28^ humoral factors, emotional factors and genetic variations,^29^ the long-term BPV has been shown to be a reproducible rather than a random phenomenon^30^ and is considered to be greatly influenced by behavioural changes (physical activity, sleep, postural changes),^31^ arterial stiffness,^32^ ^33^ dietary factors,^34^ blood lipid,^35^ poor BP control in treated patients (incorrect dosing and poor compliance with antihypertensive treatment), obesity^36^ and seasonal climatic changes.^37^ CHS comprises seven metrics—smoking, diet, physical activity, body weight, plasma glucose, cholesterol and BP, which overlaps with a number of influencing factors of long-term BPV. This might be a probable cause of the correlation observed in this study.

Previous works by our group^23^ ^38^ and several studies by other researchers have reported an inverse relationship between higher CHS and CVD incidence.^18–23^ In our group's previous investigation of individuals in the Kailuan cohort, we detected a strong inverse relationship between cumulative CVD incidence and the number of ideal health metrics at baseline.^23^ However, the probable mechanism of this association has not been fully revealed. Although arterial stiffness (represented by PWV),^39^ several CVD biomarker levels and subclinical diseases^24^ are reported to partially account for the relationship between CHS and CVD incidence, strong evidence suggested that other biological mechanisms also play important roles.^24^ Increasing evidence supports the fact that BPV, especially long-term BPV, is associated with CVD incidence.^40^ Therefore, in the light of the present results, we might speculate that a higher CHS can lead to a lower CVD incidence, and the mechanism of this favourable effect might be partly mediated by the decreased year-to-year BPV.

As is well known to all, hypertension is an essential health concern globally and has become a major health burden due to its high prevalence and associated increase in risk of CVD. According to the Chinese National Nutrition and Health Survey in 2002, Ministry of Health, the prevalence rate of hypertension was 18.8%.^41^ It is supposed that more than 200 million Chinese have elevated BP. Since the important role of BPV in BP management and CVD development has been well documented, BPV lowering has been attached more and more importance. Here, we report an inverse relationship between CHS and year-to-year BPV for the first time, which further supports that a healthier lifestyle might contribute to better BP management. This might be the novelty and significance of this study.

This study has several limitations. First, we did not strictly adhere to the health indicators proposed by AHA. Lacking specific data about diet, we adopted salt intake as a replacement, which could somewhat reduce the influence of diet on year-to-year BPV. However, salt intake in China is much higher than in other countries;^42^ therefore, it is of greater importance than other dietary indices among Chinese people. Second, the majority of participants are male, and they are notably more likely to exhibit worse health behaviours, such as cigarette smoking and poor diet. Third, we failed to obtain the detailed information about the class of antihypertensive medication. Fourth, there was a 2-year interval between BP measurements, which may impact the repeatability of the year-to-year BPV. However, a study investigating the relationship between low birth weight and year-to-year BPV used a measurement interval of 2–3 years.^43^ Additionally, the European Carotid Surgery Trial (ECST)^44^ used a 1 year interval with only one BP measurement at each visit, and their results indicated good repeatability. In this study, we performed three BP measurements during each visit, and thus our present year-to-year BPV should show better repeatability. All health examinations were performed following the same sequence, potentially reducing the impact of different seasons on BPV and thereby better reflecting the influence of different CHSs on year-to-year BPV. Last, we did not document detailed information on daily salt intake.

## Conclusions

In summary, this study detected an inverse relationship between CHS and year-to-year BPV for the first time and supported that healthier lifestyle might contribute to better BP management, which might also partly explain the inverse relationship between CHS and CVD incidence.
